# Stability of Folate and Vitamin B_12_ in Human Serum after Long-Term Storage: A Follow-Up after 13 Years

**DOI:** 10.1155/2018/9834181

**Published:** 2018-03-13

**Authors:** Eugène H. J. M. Jansen, Piet K. Beekhof

**Affiliations:** Centre for Health Protection, National Institute of Public Health and the Environment, P.O. Box 1, 3720 BA Bilthoven, Netherlands

## Abstract

In epidemiological and nutrition research, it is very important to evaluate the stability of biomarkers as function of both storage time and temperature. In this study, the stability of folate and vitamin B_12_ in human serum samples has been tested after long-term storage at −80°C up to 13 years. Serum samples of 16 individuals were used in this study. The concentration of folate and vitamin B_12_ has been determined at *t*=0 and at 1, 8, and 13 years after storage at −80°C. The folate concentrations in serum samples remained stable at −80°C. The concentration of vitamin B_12_ was decreasing during the time of the study to about 50%. The correlation of the folate and also of the vitamin B_12_ concentrations in the stored samples compared with the starting values was still good. Therefore, although the concentration of vitamin B_12_ decreased upon storage, reliable comparative analyses can still be performed.

## 1. Introduction

Folate and vitamin B_12_ are both vitamins from the one-carbon metabolism and serve as compounds that repair DNA and re-methylate homocysteine [1–3].

The folate status is associated with several chronic diseases with a large impact on public health [4]. Folate deficiency is also associated with birth defects [5]. Both vitamins are related to both cardiovascular diseases [3, 6, 7, 9] and cognition [7–10]. Therefore, they are very important in nutrition studies to assess the dietary patterns. In addition, in epidemiological and clinical research, these vitamins are often used to make risk assessments in prospective studies. In these studies, often serum or plasma samples were used from biobanks which have been stored for several years and sometimes even for decades. Therefore, the assessment of the stability of these biomarkers during long-term storage is of great importance [11].

The measurements of folate and to a lower extend vitamin B_12_ are important biomarkers in clinical medicine and in epidemiologic studies. Both vitamins can be measured by a variety of methods, but the use of autoanalyzers have become common practice [12].

In the present study, folate and vitamin B_12_ have been measured with a commercially dedicated autoimmunoanalyzer. The stability of folate and vitamin B_12_ in serum samples was determined after a long-term storage of up to 13 years at −80°C.

## 2. Materials and Methods

### 2.1. Blood Withdrawal and Storage

For the stability study, serum samples of 16 human volunteers (blood donors) were used. Samples were obtained from the Central Blood Laboratory of the Red Cross (Amsterdam, the Netherlands) with written permission of the volunteers. After blood withdrawal, serum samples were prepared within two hours, divided in aliquots, and stored at −20°C and −70°C. In this study, only samples that have been stored at −70°C were used.

**Figure 1:**
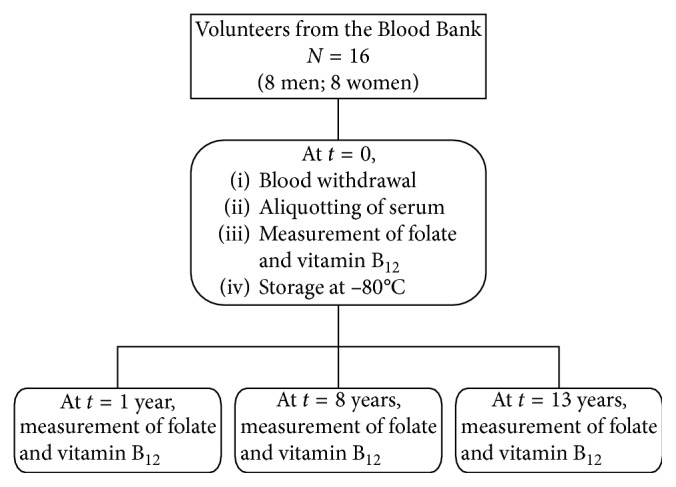
Flow diagram of the study.

The initial concentrations of folate and vitamin B_12_ at *t*=0 have been determined within 4 hours after centrifugation. For long-term stability, samples have been stored for 1, 8, and 13 years, at −70°C for one year and at −80°C for the remaining period. Samples stored at −70°C were kept in a freezer equipped with temperature recorder and sound alarm. From 1 year onwards, the samples were stored at −80°C in a freezer equipped with an automatic temperature registration system with an alarm function. In Figure 1, the flow diagram of the present study is shown.

### 2.2. Measurements of Biomarkers

The serum samples were kept on ice for less than 1 hr before the first measurements at *t*=0. The measurements were performed as a single measurement, except on day 0 (duplicate measurements). At time points 0 and 1 year, folate and vitamin B_12_ have been determined with an autoanalyzer (Hitachi 912, Roche Diagnostics, Almere, the Netherlands). A reference serum (AHSL-1) was included in each assay. During the first year of storage, the inter assay variation for folate was 1.9% (*N*=8). The inter assay variation for vitamin B_12_ was 3.4% (*N*=8).

At time points 8 and 13 years, folate and vitamin B_12_ have been determined in serum with an immunoanalyzer (Access-2, Beckman-Coulter, Woerden, the Netherlands). A pretreatment converts the folate polyglutamic acid forms to the monoglutamic acid form predominant in serum. The measurements were performed as a single measurement, with dedicated kits from Beckman-Coulter. At time point 8 years, the intra-assay variation was determined with a reference serum (Biorad number: 40192). For folate, the intra-assay variation was 1.1% (*N*=2). The intra-assay variation for vitamin B_12_ was 4.3% (*N*=2). At time point 13 years, the intra-assay variation was determined with three reference sera (Biorad-1, -2, and AHLS-2). For folate, the mean intra-assay variation was 1.6% (*N*=4). The mean intra-assay variation for vitamin B_12_ was 2.7% (*N*=4).

The statistical differences (95% confidence interval) of the values from the initial value at *t*=0 were determined with a *t*-test for two-samples assuming equal variances.

## 3. Results

At the start of the study (*t*=0), the initial concentrations of folate and vitamin B_12_ of the 16 volunteers were determined. The mean concentration for folate at *t*=0 was 6.1 ±1.7 ng/mL, and the median value was 5.8 ng/mL, with a range of 3.9–10.5 ng/mL. The mean concentration for vitamin B_12_ at *t*=0 was 471 ± 75 pg/mL, and the median value was 464 pg/mL, with a range of 381–709 pg/mL.

### 3.1. Stability of Folate and Vitamin B_12_

**Figure 2:**
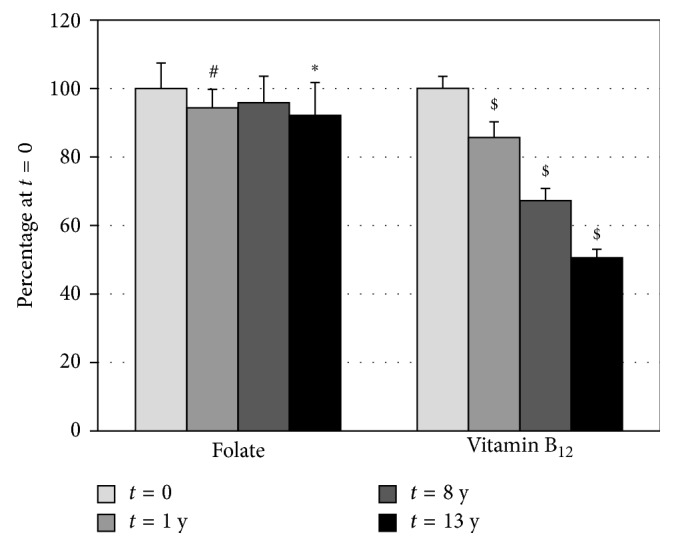
Stability of folate and vitamin B_12_ at 1, 8, and 13 years relative to *t*=0. The concentrations have been expressed as percentage of the mean value of their concentrations at *t*=0. Statistics: ^∗^ *P* < 0.05, ^#^ *P* < 0.05, and ^$^ *P* < 0.001.

The stability of both folate and vitamin B_12_ in human serum was followed during 13 years. In Figure 2, the mean values of the 16 volunteers are shown at each time point, expressed as percentage of the mean value of their concentrations at *t*=0 (before freezing of the samples).

The levels for folate are rather constant during 13 years of storage at −80°C. Somewhat lower values, but statistically significant, were observed at time points 1 and 13 years of 94.4% and 92.0%, respectively.

For vitamin B_12_, an almost linear decrease was observed during the time period of storage. The mean level of vitamin B_12_ decreased from 100% to 85.6%, 62.2%, and 50.5% at time points 1, 8, and 13 years, respectively.

### 3.2. Correlations for Folate

**Figure 3:**
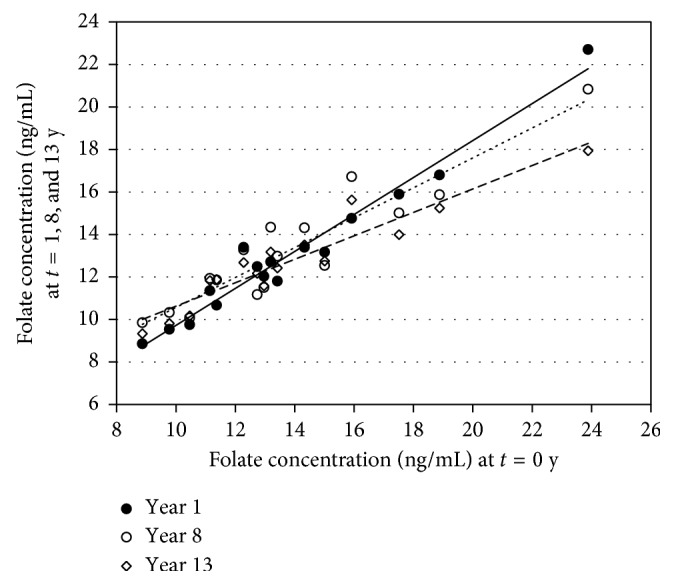
Correlation between the mean folate concentrations at *t*=0 and after storage for 1, 8, and 13 years at −70°C. The correlation lines have the following equations: *y* = 0.870*x* + 1.02 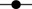, *y* = 0.703*x* + 3.53 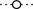, and *y* = 0.552*x* + 5.11 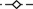 for 1, 8, and 13 years, respectively.

In Figure 3, the correlation lines of the folate concentrations of the 16 volunteers before the storage of the sera and after storage at 1, 8, and 13 years are shown. The correlations of the time points with the starting values are very good. The correlation lines have the following equations: *y* = 0.870*x* + 1.02, *y* = 0.703*x* + 3.53, and *y* = 0.552*x* + 5.11 for 1, 8, and 13 years, respectively. The correlation coefficients (*R* ^2^) were 0.961, 0.867, and 0.889 for 1, 8, and 13 years, respectively. The statistics between 2 sets of data are *P*=0.0019 for 0 versus 1 year, *P*=0.155 for 0 versus 8 years, and *P*=0.031 for 0 versus 13 years as determined with a *t*-test for equal variances.

### 3.3. Correlations for Vitamin B_12_

**Figure 4:**
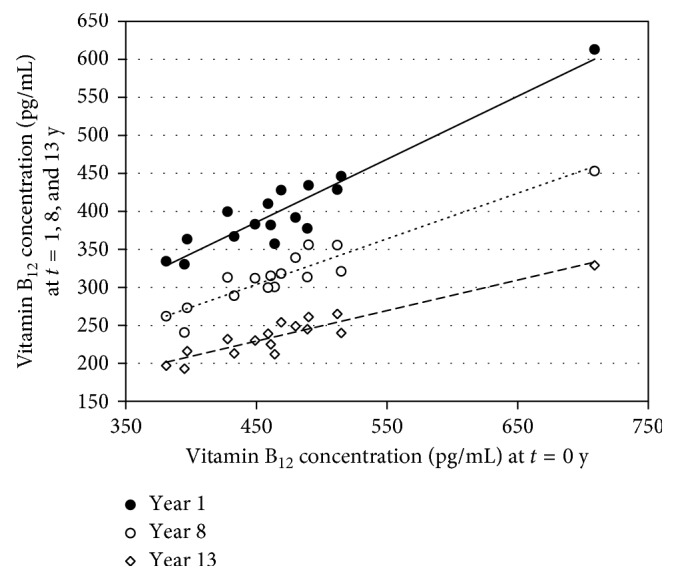
Correlation between the mean vitamin B_12_ concentrations at *t*=0 and after storage for 1, 8, and 13 years at −70°C. The correlation lines have the following equations: *y* = 0.829*x* + 2.80 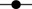, *y* = 0.600*x* + 33.7 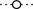, and *y* = 0.401*x* + 48.5 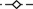 for 1, 8, and 13 years, respectively.

In Figure 4, the correlation lines of the vitamin B_12_ concentrations of the 16 volunteers before the storage of the sera and after storage at 1, 8, and 13 years are shown. Although the concentration of vitamin B_12_ decreased substantially with the duration of storage, as shown in Figure 1, the correlations of the time points with the starting values are still rather good. The correlation lines have the following equations: *y* = 0.829*x* + 2.80, *y* = 0.600*x* + 33.7, and *y* = 0.401*x* + 48.5 for 1, 8, and 13 years, respectively. The correlation coefficients (*R* ^2^) were 0.894, 0.890, and 0.865 for 1, 8, and 13 years, respectively. The statistics between the 2 sets of data are *P*=1.7∗*E* − 8 for 0 versus 1 year, *P*=1.2∗*E* − 11 for 0 versus 8 years, and *P*=3.0∗*E* − 12 for 0 versus 13 years as determined with a *t*-test for equal variances.

## 4. Discussion

Several short-term stability studies on folate and vitamin B_12_ were performed in the past [13–17]. But only one long-term stability study on folate was found in EDTA plasma for 48 months at −20°C [18]. After 6 months of storage the mean value of folate decreased to 25% of its original value, and this level remained constant until 48 months. For vitamin B_12_, only one long-term stability study was found [14] and showed a good stability during 48 months of storage in EDTA plasma at −20°C, which is a different finding with our present results.

The present study shows in which folate and vitamin B_12_ were measured after 1, 8, and 13 years of storage at −80°C that that the measurements of folate still can be done without any major change in the concentration. For vitamin B_12_, there is a consistent decrease to about 50% after 13 years of storage at −80°C. It must be noted that from 8 years on, the autoanalyzer and reagents were changed so that a possible level change could be expected, although a comparative study was performed that showed the same results for both methods. In a previous report after 1 year of storage [19], the decrease of vitamin B_12_ was already visible, but then it was not feasible to make a strong conclusion based on only one time point. Now after 13 years, it is clear that this decrease was consistent.

For epidemiological studies, however, this decrease in concentration of vitamin B_12_ has only little effect on the rank order and correlations with the starting concentrations. Rather good correlation coefficients (*R* ^2^) were found between 0.87 and 0.89 after storage. For folate, the concentrations of the stored samples remain close to the starting concentrations with good correlations with the starting concentrations; for example, the correlation coefficient (*R* ^2^) at 13 years was 0.89.

There has been one study on the long-term stability of B vitamins [20] in which storage at −25°C during 4, 6, 17, and 29 years was reported. Folate was measured as p-aminobenzoylglutamate declined at a slow rate of 0.98%/year and about 80% of the folate was recovered after 29 years of storage as determined with LC-MS/MS. For folate, p-aminobenzoylglutamate appeared to be the method of choice for the determination of folate status in stored serum samples. In our method, a pretreatment also converts the folate polyglutamic acid forms to the monoglutamic acid form which is predominant in serum.

In the same study, vitamin B_12_ status did not show any decline, as measured with a microbiological assay [20]. This is in contrast with our results. Maybe the method of choice has an influence of the outcome of the study.

In conclusion, this study shows that long-term storage of serum samples at −80°C, intended for future measurements of folate and vitamin B_12_, can still be used for data analyses, although the concentration of vitamin B_12_ decreases steadily with longer storage time.
